# Internal and External Pipe Defect Characterization via High-Frequency Lamb Waves Generated by Unidirectional EMAT

**DOI:** 10.3390/s23218843

**Published:** 2023-10-31

**Authors:** Xu Zhang, Bo Li, Xiaolong Zhang, Xiaochun Song, Jun Tu, Chen Cai, Jundong Yuan, Qiao Wu

**Affiliations:** 1 Hubei Key Laboratory of Modern Manufacturing Quantity Engineering, School of Mechanical Engineering, Hubei University of Technology, Wuhan 430068, China; zhangxu@mail.hbut.edu.cn (X.Z.); 102110151@hbut.edu.cn (B.L.); 102200031@hbut.edu.cn (X.Z.); songxc@hbut.edu.cn (X.S.); juntu@hbut.edu.cn (J.T.); 2 Wuhan Second Ship Design and Research Institute, Wuhan 430064, China; 20160069@hbut.edu.cn; 3 School of Computer Sclence And Technology, Taiyuan University of Science and Technology, Taiyuan 030024, China; yuanjundong016@tyust.edu.cn

**Keywords:** CLamb wave, high-resolution unidirectional EMAT

## Abstract

Periodic permanent magnet(PPM) electromagnetic acoustic transducers (EMATs) are commonly employed for axial defect inspection in pipelines. However, the lowest-order shear horizontal waves (SH0) guided waves have difficulties in distinctly differentiating internal and external defects. To enhance the signal-to-noise ratio and resolution, a unidirectional electromagnetic acoustic transducer (EMAT) based on Circumferential Lamb waves (CLamb waves) is developed. Through structural parameter optimization and excitation frequency adjustment, high-amplitude and low-dispersion CLamb waves are successfully generated in the high-frequency-thickness product region of the dispersion curve. Finite element simulations and experimental validation confirm the capability of this EMAT in exciting CLamb waves for the detection of crack-like defects. Experimental results demonstrate that the excitation efficiency of the CLamb EMAT exceeds that of the periodic permanent magnet electromagnetic acoustic transducer by more than tenfold. The defect reflection signal of the CLamb EMAT exhibits higher resolution and more significant amplitude compared to the PPM EMAT. The integration of this method with SH0 mode detection allows for the inspection of both internal and external defects in pipelines, offering a new avenue for EMAT applications in pipeline inspection.

## 1. Introduction

Pipelines, serving as vital transportation systems, play a pivotal role in ensuring the long-term operational safety and reliability of industrial equipment. However, the service life of pipelines is often plagued by the emergence of various internal and external defects, such as corrosion and cracks, posing significant challenges for detection and diagnosis [1]. EMAT, as a non-contact detection technique, is widely used in pipeline inspection [2]. For defects along the axis of the pipeline, circumferential guided waves have higher detection sensitivity compared to axial low-frequency guided waves. EMAT detection technology can simultaneously detect the size of pipeline cracks and the state of external coatings by exciting different transverse wave modes [3]. Wang studied the reflection and transmission coefficient curves of the circumferential SH0 wave (CSH0) and Circumferential SH1 wave (CSH1) modes with the depth of three types of axial slots through three-dimensional finite element simulation [4]. Liu used an EMAT laser for the quantitative identification of lamination defects in thin-walled metal pipes through beam analysis of lamination defects [5]. Lu analyzed the reflection of SH0-guided waves at the front and rear edges of circumferential cracks and the influence of circumferential crack geometric parameters on the two edge-reflected waves. Subsequently, the accuracy of circumferential crack range estimation was given by the time shift of the two edge-reflected waves [6]. Dixon compared finite element simulation and experiments to study the influence of inclination angle and depth of pipeline defect recesses on echo signals, improving the accuracy of electromagnetic ultrasonic defect detection through precise theoretical models and simulation models [7].

Currently, the only structure of circumferential guided wave EMAT for pipelines is PPM (periodic permanent magnet), whose excitation wavelength is adjusted by changing the period of magnets. [8]. Therefore, traditional EMATs are difficult to distinguish between internal and external defects of pipelines and difficult to significantly improve detection frequency. Furthermore, a prevalent challenge in guided wave inspection methods is the imperative need for signal enhancement to mitigate noise interference and amplify received signal amplitudes. Notably, the presence of substantial noise generated by peripheral machinery necessitates the implementation of effective countermeasures [9,10,11,12,13,14,15]. Moreover, EMAT wave detection currently mainly employs low-frequency regions, and the resolution is relatively low. High-frequency EMAT has gradually become a research highlights, and currently, high-frequency guided wave EMAT is mainly applied to thickness measurement and stress detection, greatly improving the detection resolution of EMAT [15,16,17,18,19,20,21,22,23,24,25,26,27,28]. The emergence of high-frequency guided wave EMAT provides new possibilities for improving the detection resolution of pipeline defects. Rayleigh surface waves have relatively high energy concentrated on a single surface and can propagate over long distances inside and outside solids, as well as detect defects close to the surface [29]. High-frequency CLamb waves, as a special type of circumferential Lamb waves in pipelines, have characteristics similar to Rayleigh waves, but the use of high-frequency circumferential CLamb waves for pipeline defect detection has not been well studied.

For this reason, this paper improves the traditional Rayleigh wave structure of EMAT and, through a suitable excitation frequency, can excite the circumferential high-frequency CLamb wave mode with a relatively high signal-to-noise ratio in the circumferential direction of the pipeline. Its characteristics and rules of interaction with internal and external defects of the pipeline are studied and compared with the detection results of traditional PPM EMAT to realize distinction and high-resolution detection of internal and external defects of pipelines.

## 2. Principle and Design of EMAT

**Figure 1:**
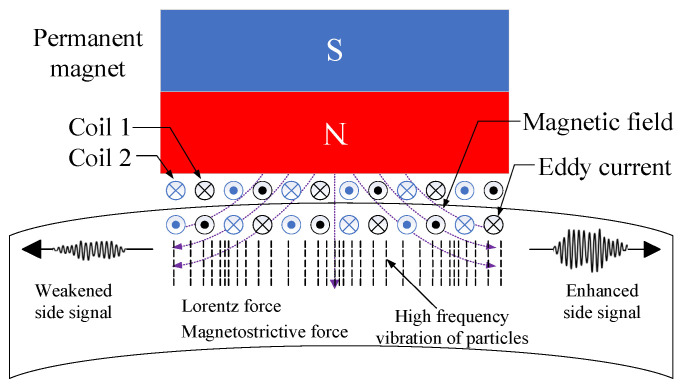
A schematic diagram of a unidirectional high-frequency CLamb wave EMAT.

Common EMAT probes are mainly composed of permanent magnets or electromagnets, coils, and test pieces. The circumferential guided waves excited by traditional EMATs propagate along both sides of the pipeline, and the signals on the far side will interfere with the detection of defects. To solve this problem, research and application of unidirectional surface wave electromagnetic acoustic transducers (EMATs) have become relatively mature [30,31]. In addition, unidirectional propagation can also significantly improve the detection capability and signal-to-noise ratio of ultrasonic signals [32,33]. Therefore, the EMAT used in this paper is shown in Figure 1. The traditional PPM EMAT is used to excite SH-guided waves. However, CLamb is a guided wave mode formed by longitudinal and shear vertical waves (SV waves) propagating along a pipe of limited thickness through multiple reflections and refractions. Its excitation method is similar to Lamb waves, requiring periodic radial or circumferential force sources. When the pipeline is a ferromagnetic material, the working principle is mainly Lorentz force and magnetostrictive force. Since both the Lorentz force and the magnetostrictive force are in the same direction and parallel to the surface of the pipe, the superposition of the two principal forces significantly increases the transducer efficiency. Additionally, the operating frequency can be adjusted by adjusting the coil spacing.

## 3. Finite Element Analysis of High-Frequency CLamb Waves

**Figure 2:**
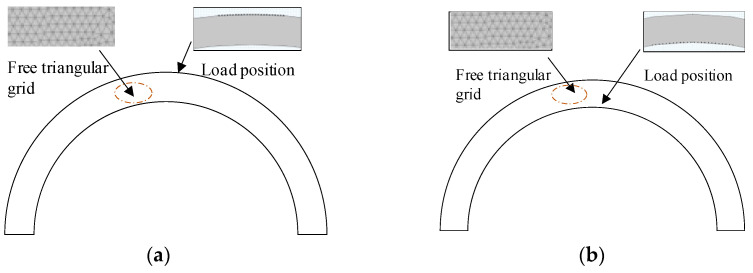
Simulation model of high-frequency CLamb wave excitation. (**a**) External excitation of the pipe and (**b**) internal excitation of the pipe.

To study the rules of interaction between unidirectional CLamb waves and defects inside and outside the pipeline, a two-dimensional finite element simulation model, as shown in Figure 2, is established. The thickness of the iron pipe in the model is 6 mm, and the diameter is 220 mm. A rectangular groove with a depth of a is set on the surface of the pipe, with a constant width of 1 mm. The groove is perpendicular to the axis of the pipe. The Young’s modulus E of the iron pipe is 200 GPa, the Poisson’s ratio is 0.29, and the density is 7850 kg/m^3^. In the simulation process, a simplified force source model is used to replace the coupled multi-field model to improve the calculation speed. Twenty-four loading points are set separately for detection on the outer side and inner side of the pipe, with an interval of 1 mm between each loading point consistent with the actual coil. To ensure the accuracy and speed of simulation calculation, the size of the grid unit is 1/10~1/20 of the ultrasonic wavelength. Since the wavelength is 2 mm, the maximum grid unit is 0.2 mm. According to the actual situation of electromagnetic ultrasonic detection experiments, the coil excitation frequency is set to 1.45 MHz in the model, and a Hanning window with a modulation period of six is used to modulate the cosine signal. The excitation function is shown in Equation (1):

(1) $$\left\{\begin{array}{c} y=0.5A(1-\cos\frac{2\pi ft}{n})\cos2\pi ft,0<t\leq nT\\ 0,t>nT \end{array}\right.$$

where *A* is the amplitude of the excitation current, *T* is the excitation period, equal to the pulse width, and *n* is the excitation current period, taking 5.

**Figure 3:**
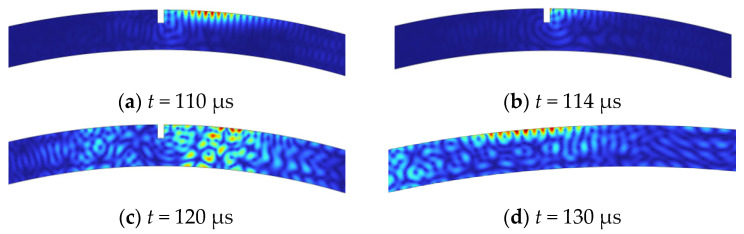
Simulation results of propagation characteristics of high-frequency Lamb waves excited on the outer side of the pipeline and interact with the defect at different times (*t*).

Taking the external excitation of the pipeline as an example, the displacement distribution snapshots at different times are shown in Figure 3. The results show that a unidirectional propagating high-frequency CLamb wave can be excited using an approximate point source model, with characteristics similar to Rayleigh waves. When the CLamb wave interacts with the defect, part of the energy returns from the defect in the form of a reflected wave of the same mode. Therefore, high-frequency CLamb waves can be used to detect near-surface defects.

**Figure 4:**
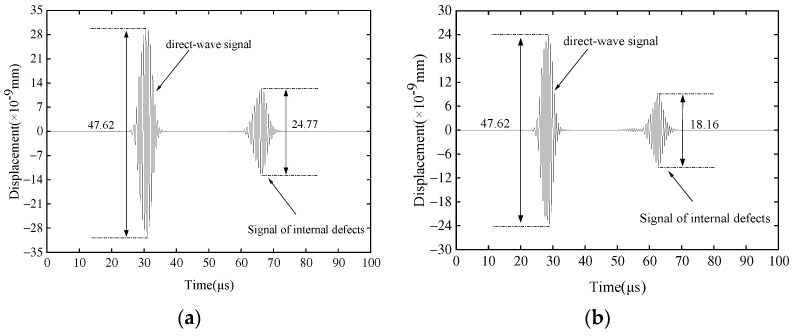
Simulation results of radial displacement at the observation point. (**a**) Received signal outside the pipe; and (**b**) received signal inside the pipe.

To avoid the overlap of signals, the distance between the observation point and the defect should be set sufficiently large. The internal defect and external defect positions are set separately, 300 mm away from the excitation end. In the detection on the outer side and inner side of the pipeline, the excitation point and receiving point are set on the same side of the defect, and the receiving point and excitation point are both 200 mm away. The displacement of points at different positions is extracted and analyzed separately. Figure 4 shows the out-of-plane displacement at the observation point, with a center frequency of 1.45 MHz and a defect depth a = 2 mm. The first wave packet in Figure 4a is the direct wave on the outer side of the pipeline, corresponding to Figure 2a, with an amplitude of 47.62 × 10^−9^ mm, and the second wave packet is the reflected wave, with an amplitude of 24.77 × 10^−9^ mm. The first wave packet in Figure 4b is the direct wave at the observation point inside the pipeline corresponding to Figure 2b, with an amplitude consistent with the outer side of the pipeline, and the second wave packet is the reflected wave, with an amplitude of 18.16 × 10^−9^ mm. To reduce the number of meshes and improve computational efficiency, the simulation model includes only half a pipe and sets absorption boundaries at the end faces, so there are no signals of weakened side direct waves shown in Figure 4.

For groove defects in pipelines, once the length is settled, the main geometric parameter is depth. To avoid the influence of the incident wave amplitude on the analysis of the relationship between the received signal and the defect depth, the reflection coefficient is selected instead of directly using the amplitudes of each wave as the dependent variable of crack depth; that is, the ratio of the reflected wave amplitude to the incident wave amplitude.

Equation (2) represents the expression for the reflection coefficient.

(2) $$R=\frac{A_{r}}{A_{I}}$$

where *A_r_* is the amplitude of the reflected wave, and *A_I_* is the amplitude of the incident wave. For example, in Figure 4a, the reflection coefficient is calculated by 24.77/47.62.

**Figure 5:**
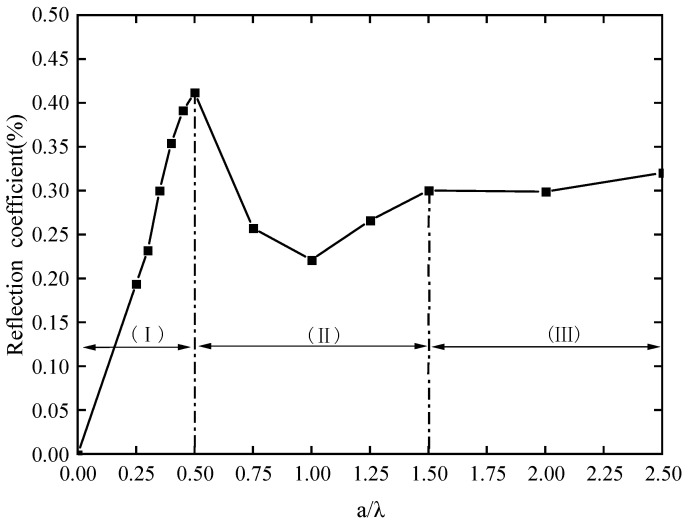
Reflection coefficient change curve with a/λ.

Under the same excitation conditions, the trend of changes in the reflection coefficient is obtained by parameter scanning the crack depth, as shown in Figure 5. It can be seen from the figure that the relationship between the reflection coefficient and a/λ (the ratio of defect depth to wavelength) shows a non-monotonic nature. When the depth of the defect increases to half of the wavelength, the reflection wave shows an upward trend with the increase in defect depth in this interval, indicating that the deeper the defect, the easier it is to reflect the surface wave back. When the depth of the defect is greater than half of the wavelength, the reflection coefficient initially shows an oscillating state, then enters a gentle state, and the reflection coefficient basically does not increase.

## 4. Experiments

### 4.1. Experimental Platform

**Figure 6:**
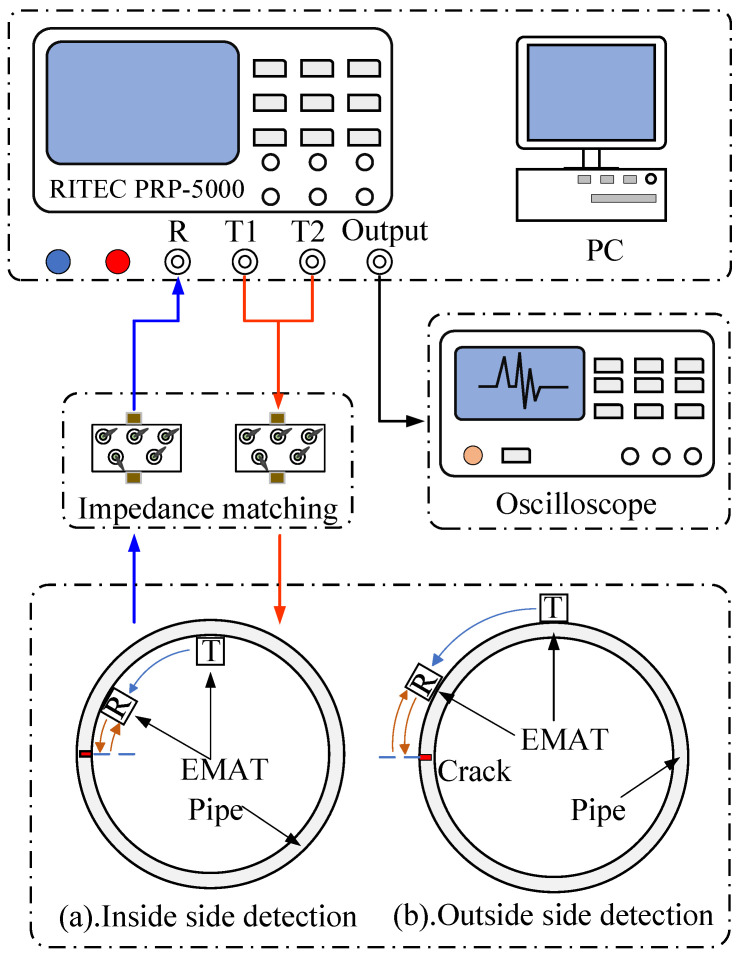
Experimental system platform. (**a**) Layout diagram of EMAT on the outer side of the pipeline; and (**b**) layout diagram of EMAT on the inner side of the pipeline.

The experimental system platform is shown in Figure 6, consisting of an oscilloscope, Ritec-5000 high-energy pulse transmitter/receiver, test iron pipe, and the developed electromagnetic ultrasonic sensors.

In the experiment, an iron pipe with a diameter of 270 mm and a wall thickness of 6 mm was used as the detection object, as shown in Figure 6. Three grooves of different depths were fabricated on the inner and outer sides of the iron pipeline, where the inner defect and outer defect were not at the same place along the pipeline. The depths of the grooves were 2 mm, 3 mm, and 5 mm, respectively, which were consistent with the groove dimensions used in the simulation model. The spacing between all grooves was 50 mm. In this platform, Ritec-5000 generates a Tone-burst signal and receives the induced voltage signal. Through adjustment of the impedance matching network, this signal excites the electromagnetic ultrasonic transducer to generate a unidirectional CLamb wave. After propagating a certain distance, the CLamb wave is captured by the receiving EMAT and amplified by the amplifier in the Ritec-5000 high-energy pulse transmitter/receiver. After filtering processing by a bandpass filter, it is displayed on the oscilloscope. Figure 6a,b show the same type of electromagnetic acoustic transducer (EMAT) outside and inside the pipe layout diagram. In the figure, the light blue lines represent direct waves, while the brown lines represent reflected waves after encountering defects. The red arrow indicates the transmission path of the excitation signal, while the blue arrow indicates the transmission path of the received signal. For comparison purposes, the position of the excitation and receiving transducers remained unchanged in all subsequent experiments, and they were 200 mm apart both inside and outside the pipe.

**Figure 7:**
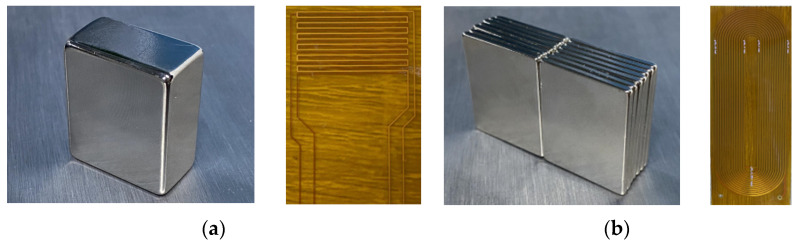
Physical drawings of two EMATs. (**a**) CLamb wave EMAT; and (**b**) PPM EMAT.

The sizes of magnets used for the CLamb wave EMAT and the PPM EMAT are as follows: the size of the large magnet is 30 mm × 25 mm × 14 mm, while the size of the small magnet is 30 mm × 25 mm × 2 mm. The number of magnets used for PPM EMAT is 7 pieces on one side, totaling 14 pieces on both sides, with a volume twice that of the CLamb wave EMAT magnet. The magnetization direction faces the 30 mm height direction. Flexible circuit boards (FPC) were used in the design of the meandering coil and runway coil. Both types of coils adopted a double-layer structure. The meandering coil includes 12 turns of wire, with each wire 20 mm in length and 0.2 mm in width, with 1 mm spacing between adjacent wires. The runway coil includes 0.2 mm wide wires 30 mm in length, with a total of 26 turns of wire. Photos of the two types of EMATs are shown in Figure 7.

### 4.2. Selection of Excitation Frequency

**Figure 8:**
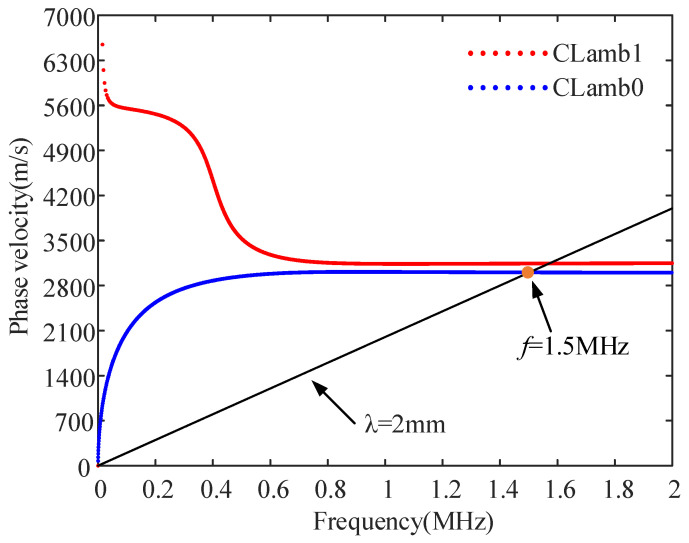
Phase velocity dispersion curves of the iron pipe.

Based on the pipe parameters used in the simulation, the phase velocity dispersion curve of the sample was calculated, as shown in Figure 8. The frequency is selected considering that the frequency dispersion curve in the band is as flat as possible, so it needs to be greater than 1.2 MHz. Theoretically, the coil spacing can be further reduced to increase the excitation frequency; however, in general, the higher the frequency, the greater the wave attenuation. Considering EMAT bandwidth, the optimal excitation frequency was determined to be 1.5 MHz. With the designed coil spacing of 1 mm, the optimal excitation wavelength was 2 mm. Considering the potential discrepancy between actual experiments and theoretical calculations, the optimal frequency was determined to be 1.45 MHz, near the theoretically calculated value of 1.5 MHz through frequency sweeping experiments.

**Figure 9:**
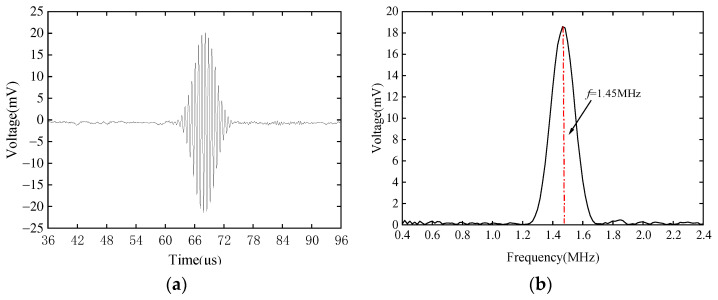
Time domain and frequency domain signal diagrams of CLamb wave for (**a**) time domain and (**b**) frequency domain.

The CLamb wave is excited and received on an intact pipeline, and the time domain and frequency domain diagrams of the received signal are shown in Figure 9. It can be seen from the figure that with no defects, the EMAT used in this experiment can better excite high-frequency Lamb waves with small dispersion and a high signal-to-noise ratio, which can theoretically achieve unidirectional propagation. Figure 9 mainly focuses on the time domain signal and frequency domain signal of the direct wave of the enhanced side in practical experiments. In fact, the complete data from this signal contain the signal on the attenuating side, but to make the excitation signal on the enhancement side more clear, the signal on the weakened side is not included in the interception time range.

### 4.3. CLamb EMAT Defect Detection Experiment

**Figure 10:**
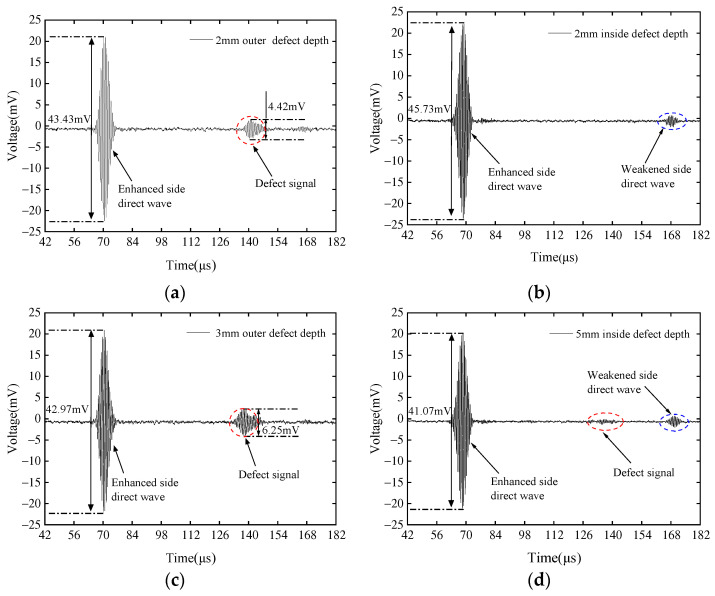

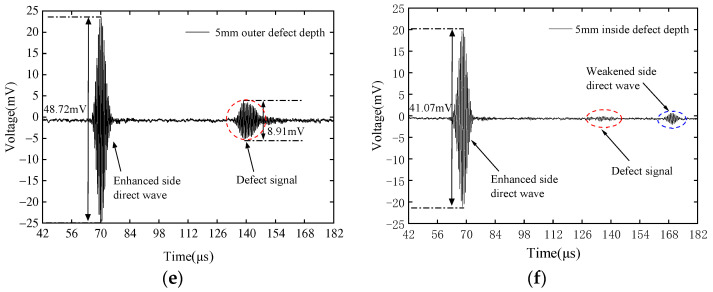
Comparison of the signals when the EMAT is arranged on the outer side of the pipeline and the depths of the external defects are (**a**) 2 mm, (**c**) 3 mm, and (**e**) 5 mm, with the signals when the depths of the internal defects are (**b**) 2 mm, (**d**) 3 mm, and (**f**) 5 mm, respectively.

By utilizing the dual output channels and output delay function of Ritec-5000 to generate 1.45 MHz signals with a phase difference of 90° acting on the meandering coil, the CLamb wave EMAT generates ultrasonic waves propagating unidirectionally, and the position of the transducer and defects is shown in Figure 7a. Three groups of defects with the same depth were set inside and outside the pipeline. The depth-to-wavelength ratios a/λ were 1, 1.5, and 2.5. Figure 10 shows a comparison of the detection results for the three external defects and three internal defects of the pipeline with the EMAT arranged on the outer side of the pipeline.

**Figure 11:**
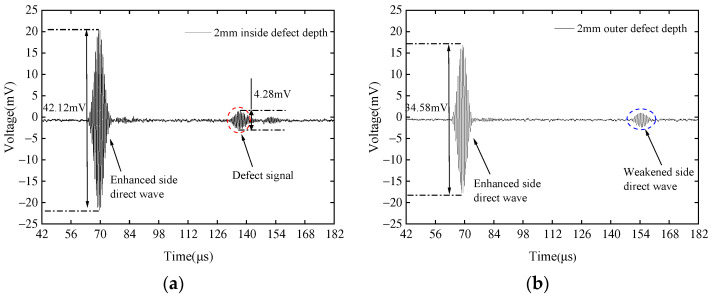

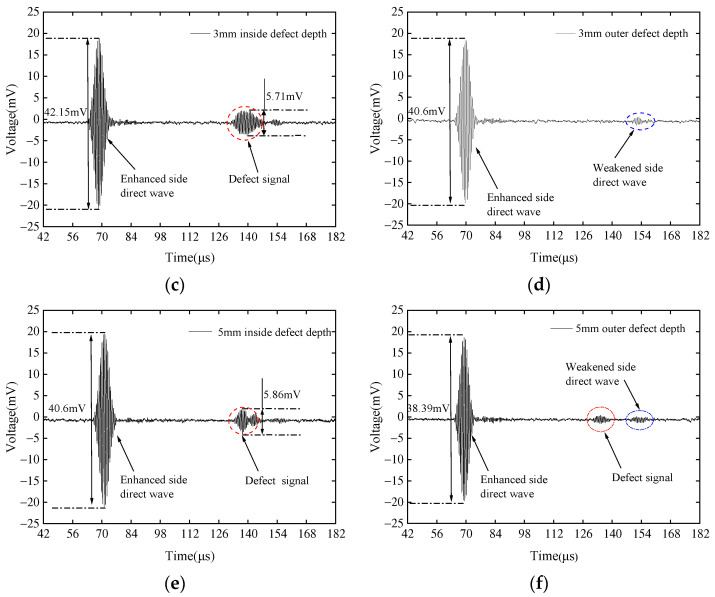
Comparison of the signals when EMAT is placed on the inner side of the pipe and the depth of the internal defects is (**a**) 2 mm, (**c**) 3 mm, and (**e**) 5 mm, and the depth of the external defects is (**b**) 2 mm, (**d**) 3 mm, and (**f**) 5 mm, respectively.

It can be clearly seen from Figure 10a,c,e that for defects on the same side as the EMAT, there are obvious reflected waves, the direct wave on the attenuated side disappears, and the trend of change in reflection coefficient is close to simulation, i.e., it increases with increasing depth. By comparing ace with Figure 10b,d,f, it can be seen that even at the same depth, the reflection characteristics of internal and external defects are significantly different. No reflection signal is detected in Figure 10b,d, only the direct wave on the attenuated side, while in Figure 10f, a low signal-to-noise reflected wave can be seen due to the greater defect depth. Therefore, the CLamb waves excited by the external EMAT of the pipeline are only sensitive to external defects of the pipeline, and the appearance of defect reflection waves can determine that the defect is located on the outer side of the pipeline. Similarly, when the EMAT and defect positions are as shown in Figure 7b, they are placed on the inner side of the pipeline. Three groups of defects with the same depth were set inside and outside the pipeline, with depth-to-wavelength ratios a/λ of 1, 1.5, and 2.5. Figure 11 shows the detection results for external defects and internal defects with the EMAT arranged on the inner side of the pipeline.

It can be clearly seen from Figure 11a,c,e that there are obvious reflected waves when detecting internal defects of the pipeline, and the trend of change in reflection coefficient is close to simulation, i.e., it increases with the increase of depth. The attenuated direct wave signal disappears. By comparing Figure 11a,c,e with Figure 11b,d,f, it can be seen that an attenuated direct wave can be received in all figures. Even at the same depth, the reflection characteristics of internal and external defects are significantly different. No reflection signal is detected in Figure 11b,d, only the attenuated direct wave, while in Figure 11f, a low signal-to-noise reflected wave and attenuated direct wave can be seen due to the greater defect depth. In Figure 10a,c,e and Figure 11a,c,e, the weakened side direct waves are not clear because the signal of the weakened side is the same as the nature of the enhancement side, but the amplitude is very small, resulting in a decrease in transmission energy of the weakened side signal after passing the defect signal from the far end.

### 4.4. Defect Detection by PPM EMATt

**Figure 12:**
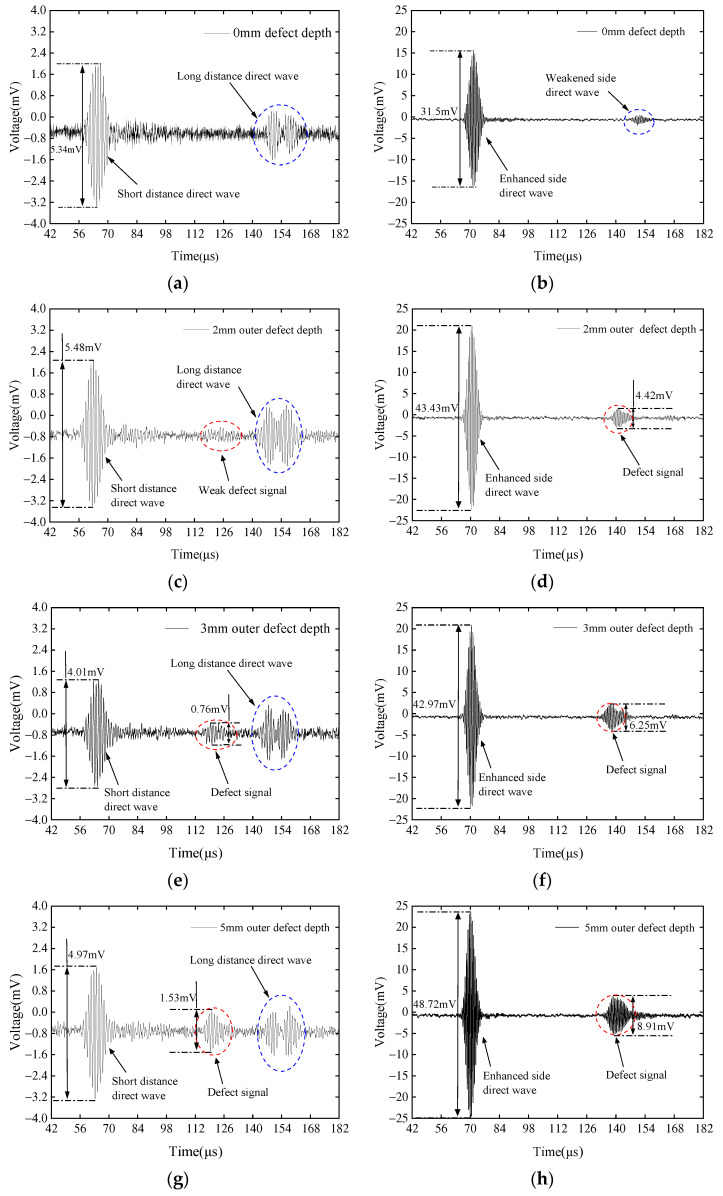
Comparison of the signals when using PPM EMAT to detect external defects with depths of (**a**) 0 mm, (**c**) 2 mm, (**e**) 3 mm, and (**g**) 5 mm and using CLamb wave EMAT to detect external defects with depths of (**b**) 0 mm, (**d**) 2 mm, (**f**) 3 mm, and (**h**) 5 mm, with the EMAT placed on the outer side of the pipeline.

Taking the inner side of the pipeline as an example, PPM EMAT was used to replace CLamb wave EMAT for detecting the same defects. The physical map of the magnet is shown in Figure 6b. The speed of the shear wave in iron is about 3100 m/s, and the optimal excitation frequency can be calculated as 775 kHz according to the relationship between wave speed and frequency and according to wavelength. The actual optimal excitation frequency is tested by experiments and set as 773 kHz. Due to the narrow magnet width, which would cause difficulties in magnetization and low magnetic flux density, this experiment did not adopt the same wavelength design as the CLamb wave EMAT; the magnet width was only reduced to 2 mm, corresponding to a wavelength of 4 mm. This width and excitation frequency are higher than the commonly used excitation frequency of PPM EMATs in literature. When the EMAT was placed on the outer side of the pipeline, Figure 12 compares the reflected wave signals of defects with different depths detected by the PPM EMAT and the CLamb wave EMAT.

Figure 12a,b are the received signals of the two types of waves with no defect, which are used as reference signals. The resolution of ultrasonic waves is wavelength-dependent, so the smaller the wavelength, the higher the resolution. The wavelength of the CLamb wave can be independent of magnet width, much smaller than the commonly used PPM structure EMAT excitation-guided wave wavelength, so the resolution is higher. As can be seen from Figure 12a,b, when the defect depth is 2 mm, the PPM EMAT can hardly detect the defect; however, the CLamb wave EMAT can show the defect signal clearly. The detection resolution of high-frequency CLamb waves is significantly higher than CSH0 waves, with smaller signal dispersion and a higher signal-to-noise ratio. Although the reflection coefficient of CLamb waves is significantly lower than CSH0 waves, with a magnet volume of only half of the PPM structure magnet, the amplitude of the reflected wave is significantly higher than the CSH0 wave. The comparison of the two EMATs on different depths of defect is listed in Table 1. Since Figure 12c shows a very weak defect signal, only depths of 3 mm and 5 mm are selected to compare the performance of the two EMATs.

From the data in Figure 12 and the calculations in Table 1, it can be found that when the defect depth is greater than 2 mm, the amplitude of the direct wave signal of the CLamb wave EMAT is close to 10 times that of the PPM EMAT, and the amplitude of the defect echo signal is 8.22 and 5.82 times that of the PPM EMAT, respectively.

## 5. Conclusions

In this article, a novel high-frequency CLamb wave method for the detection of a crack-like defect is proposed by experiment. The high-frequency CLamb wave is similar to Rayleigh surface waves and is more sensitive to the near-surface defect. The unidirectional EMAT is designed to excite a high-resolution, low-dispersion CLamb wave. The simulation and experiment results show that the designed EMAT can excite a pure high-frequency CLamb wave and is effective in detecting cracks on the same side. Through the comparison experiment of detecting defects with the same depth inside and outside the pipeline, it can be seen that CLamb waves can effectively distinguish between internal and external defects of the pipeline. The reflected waves of defects with the same depth can be clearly distinguished from the reflection characteristics due to the different positions of defects inside and outside the pipeline. The CSH0 wave currently used cannot clearly distinguish the position of defects due to its symmetric distribution of energy along the pipeline direction. Therefore, the supplementary detection of CLamb waves can effectively distinguish between internal and external defects on the basis of CSH0 detection.

CSH0 waves are usually excited by PPM magnets. Increasing the frequency requires thinning the magnet, which will significantly reduce the excitation efficiency. For the CLamb wave EMAT with a magnet quantity of only half of the PPM magnet and the excitation frequency nearly double that of the PPM EMAT, the amplitude of the excited CLamb wave can reach ten times that of the PPM EMAT. Although the reflectivity of CLamb waves is far lower than CSH0 waves due to its extremely high excitation efficiency, the amplitude of its reflected waves for defects with the same depth is still significantly higher than CSH0 waves. Furthermore, due to its higher resolution, its ability to detect small defects is higher than PPM-type EMATs. In the future, further research will be carried out on the optimization method of CLamb wave EMATs as well as the quantification and imaging of defects.

## Figures and Tables

**Table 1:** The comparison of the two EMATs on different depths of defect.

Defect Depth	Direct Wave Amplitude (CLamb Wave EMAT/PPM EMAT)	Direct Signal Amplitude (CLamb Wave EMAT/PPM EMAT)
3 mm outer defect	42.97/4.01 = 10.72	6.25/0.76 = 8.22
5 mm outer defect	48.72/4.97 = 9.80	8.91/1.53 = 5.82

## Data Availability

Not applicable.
